# Clarification to Commentary: Acute Effects of Exercise Mode on Arterial Stiffness and Wave Reflection in Healthy Young Adults: A Systematic Review and Meta-Analysis

**DOI:** 10.3389/fphys.2020.00257

**Published:** 2020-03-24

**Authors:** Doris R. Pierce, Kenji Doma, Anthony S. Leicht

**Affiliations:** ^1^Sport and Exercise Science, James Cook University, Cairns, QLD, Australia; ^2^Sport and Exercise Science, James Cook University, Townsville, QLD, Australia

**Keywords:** vascular, augmentation index, arterial stiffness, pulse wave velocity, opinion

## Introduction

We read with great interest the commentary and concerns raised by Kingsley and Tai (2019) regarding the proposed inaccurate use of data from their manuscript (Tai et al., 2018) in our systematic review and meta-analysis (Pierce et al., 2018). We were surprised to read this commentary and raised concerns given that the corresponding author (Dr. Kingsley) was contacted during the development of our manuscript with his response included into the review.

### Concern 1: Incorrect Data

The values that were used in our review (Pierce et al., 2018) were indeed different to the published data (Tai et al., 2018) but not, as suggested by Drs. Kingsley and Tai, because they were re-calculated by us. We noted during data extraction that the values for augmentation index (AIx, %) published in Table 1 (Tai et al., 2018) were markedly different to AIx (%) values reported for many other studies. To clarify the matter, the lead author (DP) emailed the corresponding author, Dr. Kingsley, on January 2 2017, questioning if the values may have possibly been normalized or may have been subject to a typographical error, as they were so different to all other reported values. The corresponding author replied by email on January 6, 2017 as follows:

“The AIx that is reported has not been normalized to pulse pressure. Interesting despite 4 reviewers and nobody noticed. Regardless, the output doesn't change. The groups are still significantly different across time.

Control: Rest: 11.9 ± 19.5, Recovery: 9.8 ± 10.5
ARE: Rest: 12.5 ± 6.0, Recovery: 19.0 ± 11.9
*F* = 10.296, Eta = 0.193, *p* = 0.003”

Consequently, these values were used in our systematic review and meta-analysis including figures. We were somewhat confused that the author didn't recognize the data or remember providing it to us for this review. Our Methods section clearly stated that “Corresponding authors were contacted where data were not clear or unavailable and data were updated for inclusion into this review.” The implication that we have misrepresented someone else's data is distressing and one we refute.

### Concern 2: The Authors Were Not Contacted

As highlighted above, the corresponding author was indeed contacted and his response included into the review/meta-analysis.

## Discussion

Overall, the concern that we have re-calculated data from prior work, incorrectly, and then used it in the meta-analysis, was unsupported. We stand behind the integrity of our review and look forward to clarification of this matter.

## Author Contributions

All authors listed have made a substantial, direct and intellectual contribution to the work, and approved it for publication.

### Conflict of Interest

The authors declare that the research was conducted in the absence of any commercial or financial relationships that could be construed as a potential conflict of interest.
